# Ten simple rules for coordinating a large digital health project: Perspectives from EU and implications for global contexts

**DOI:** 10.1371/journal.pcbi.1013957

**Published:** 2026-02-23

**Authors:** Lucia Sacchi, Blaž Zupan, Silvana Quaglini

**Affiliations:** 1 Department of Electrical, Computer, and Biomedical Engineering, University of Pavia, Pavia, Italy; 2 Faculty of Computer and Information Science, University of Ljubljana, Ljubljana, Slovenia; 3 Department of Education, Innovation and Technology, Baylor College of Medicine, Houston, Texas, United States of America; Dassault Systemes BIOVIA, UNITED STATES OF AMERICA

## Abstract

Coordinating a large-scale digital health project requires a unique mix of scientific leadership, administrative skill, and human sensitivity. Drawing from our experience leading CAPABLE, a European Horizon 2020 project aimed at improving the quality of life of cancer patients through AI and telemedicine, we present ten practical rules for navigating the complex landscape of multi-partner biomedical research. These rules address challenges such as building balanced consortia, managing timelines and regulatory requirements, ensuring cultural alignment, and promoting long-term impact through dissemination and exploitation. The paper specifically addresses international research projects at the intersection of healthcare and IT and their peculiar challenges, typically connected to the interplay of different actors such as academics, healthcare personnel, and industry partners located in different countries, each from diverse backgrounds and different working practices. Our goal is to provide researchers and project coordinators with concrete guidance to increase the likelihood of success in future large digital health initiatives.

## Introduction

The calls for medium to large research and development projects in the area of biomedicine and data science have become increasingly common today. These initiatives are designed to address complex scientific, technological, and societal challenges, often requiring multidisciplinary expertise and strong coordination. Usually bringing together 5–20 partners, such as universities, companies, and start-ups, with budgets ranging from 1 to 15 million euros, these projects require substantial effort in their planning and coordination.

Existing literature on the coordination of large-scale digital health projects highlights the importance of governance structures, stakeholder alignment, and sustainability planning in complex consortia [1–5]. However, much of this work focuses on specific project types or governance frameworks, while practical guidance on building and managing multidisciplinary collaborations in healthcare IT remains limited.

Over the years, the authors of this paper have participated in and managed several collaborative projects funded by the European Union (see, for example, [6–8], gaining valuable insights into their management, and they had experience of working within large consortia, managing time, deliverables, milestones, and non-trivial reporting to the EU Commission. Therefore, the perspectives presented in this paper primarily reflect the context of EU-funded projects. Nevertheless, many of the lessons learned are broadly applicable to international research initiatives, especially those conducted at the intersection of healthcare and information technology. Such projects are inherently complex, not only because of their interdisciplinary nature, but also because they involve professionals from diverse backgrounds, each with distinct working practices and priorities.

In this paper, we share the key lessons learned from our extensive experience, while concrete examples are taken from our recent CAPABLE project. (CAncer PAtients Better Life Experience), funded under the Horizon2020 program, and aimed to improve the quality of life of cancer patients, by exploiting big data, artificial intelligence, and telemedicine [7]. The project lasted 49 months, starting on January 1st 2020 and ending on January 31^st^ 2024, and involved 12 partners from 7 countries (Italy, Spain, Poland, The Netherlands, the United Kingdom, Israel, and Lithuania). The consortium comprised 5 universities, 3 small and medium-sized enterprises, 1 large company, 3 hospitals, and 1 patients’ association. The team included 32 people, 17 females (53%) and 15 males (47%). The total budget allocated to the project was approximately €6 million. We discuss the decisions and strategies that helped us overcome challenges, build strong collaborations, and achieve a successful outcome.

We believe that the strength of a good management relies in two complementary aspects: being firm enough to maintain direction (ensuring that everyone follows a unified path) while fostering a calm and collaborative atmosphere. Achieving these two goals is not easy, but it is essential for successful project execution. So, in this paper, we share practical insights that may assist other project leaders, involved in managing interdisciplinary biomedical projects, achieving these goals.

The article was formulated as a series of rules intended for the project leader, who has the authority to direct the various design and organizational choices.

## Rule 1: Assembling the right team

**Fig 1:**
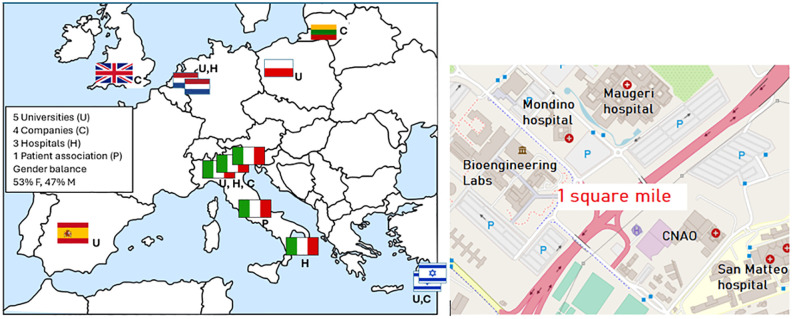
Characteristics of the CAPABLE consortium. Left: the consortium is balanced in terms of the geographical distribution of partners, their technical and biomedical expertise, and gender (53% females, 47% males). Right: An example of effective collaboration enabled by physical proximity in Pavia, Italy. The image depicts the “biomedical square mile,” where the engineering faculty is located near three research hospitals and CNAO, a leading center for hadron therapy.

Choosing the right collaborators is crucial. It is advisable to start building the team exploiting long-term collaborations, so that everybody’s skills and weaknesses are known beforehand. Once the main partners are chosen, match-making facilities offered by funding bodies [9] may be useful to address specific gaps, which can remain because a good consortium implies balance in many respects. It must include geographically dispersed countries, public and private institutions, large industries and emerging spin-offs, and it must achieve gender equity (Fig 1, left panel). The geographical distribution of partners may also affect the efficiency of collaboration. We realized that medical partners in charge of conducting clinical studies benefit from having a nearby technical partner, since possible technical problems are easier to solve in presence (Fig 1, right panel). Moreover, consider that technical partners must collaborate with the Information Technology (IT) teams of the hospitals, starting from the initial phase of software requirements collection, until the clinical study deployment. Another lesson learned is the need to include experts in ethics and regulatory affairs, such as an attorney or legal counsel, because you will need them from the beginning. In EU projects, for example, as soon as the proposal is approved, the Project Coordinator (PC) must collaborate with the other partners’ grants and legal offices to coordinate the preparation of two critical documents: the Grant Agreement (GA), i.e., the “contract” between the coordinator and the EU, and the Consortium Agreement (CA), i.e., the “private rules” governing intellectual property right and interactions among partners. The preparation of contractual and administrative documents often falls outside the expertise of the project leader, who is typically experienced in research, but may lack practical knowledge and writing skills in the area of regulations and legal rights. Since negotiations between administrators and lawyers can be tense and challenging, legal support is essential.

## Rule 2: Hire a skilled Project Manager

As project leader, you will be the primary guide of the project in all aspects, from writing an excellent proposal, to building an outstanding consortium, negotiating the budget with partners, and coordinating the scientific activities. The leading institution is responsible for all communication with the funding agency. For EU projects, the EU Commission assigns a project officer (PO), an administrator who controls that the project runs smoothly and meets the deadlines. POs may change during the project. In CAPABLE, we had POs from different backgrounds (medicine, engineering, biology, health policy, economics), and it has been important to be prepared to communicate with someone who may not fully understand the technical details of the project. Managing all this administration and keeping track of deadlines, deliverables, and milestones is a huge task that can overwhelm the project leader, especially if, as already mentioned, they are more experienced in research than in administrative tasks. If your institution doesn’t have dedicated staff, it is very useful to hire a Project Manager (PM) to help you handling the administrative and financial tasks throughout the duration of the project. Table 1 shows how the subjects of all the messages exchanged with the PM of CAPABLE, collected through the Basecamp project management platform (https://basecamp.com/), were classified into nine categories. Most of the communication was about the administration and management of the project and not about the content.

**Table 1:** The most frequent Project Manager’s activities (as from the CAPABLE experience).

Managing deadlines for scientific deliverables and financial reports
Defining dates for consortium meetings and project reviews with the funding agency
Defining dates for periodical online meetings (typically WP meetings)
Define and manage project management tools
Guide the formulation of amendments to the grant agreement or informal agreements among partners
Keep track of dissemination and exploitation activities
Prepare and consolidate financial reports
Manage the interaction with the funding agency
Manage the website at the funding agency

The PM must be familiar with the funding agency’s administrative protocols, and with the budgeting rules. While undesirable, budget shifts are very likely to arise in large consortia and over extended projects, as exemplified by the scenarios discussed in Rules 4 and 5. Finally, the PM will be your lifeline in your interactions with the agency website. As the project leader, you should have regular and frequent meetings with the PM, as you are two of the most important figures in the project, and you are in charge to prevent/manage problems or negotiations. And any large project will have these in abundance.

## Rule 3: The principal investigator is the proponent and must drive the project

As the project leader, ensuring that the entire project consortium breathes as one is an important part of your role, and support from partners in this regard may vary. This becomes clear already during the proposal-writing phase: while everyone is generally enthusiastic about joining the consortium, the low acceptance rates (e.g., 11.9% for Horizon 2020 [10]) tend to make them cautious in how much time and energy they invest. As a result, many partners concentrate mainly on their own assigned tasks, and you may occasionally need to remind them or encourage broader engagement. Once the project is funded, everyone should be committed to work on their tasks, but problems and skepticism may persist, and you must be ready to push when you perceive delays or loss of motivation. You are the only one who oversees the entire project and cares that everything is tight together neatly. You must adjust your schedule to prioritize the project over other duties, because “your” project is the most important thing you do for its entire duration, while the rest of the partners may have other more important items on their daily schedules. For example, bioengineers often complain about the (almost apparent) poor participation shown by some of the involved physicians, while these will rightfully claim that their job is to care for patients, and hospitals are time and task-critical environments, where physicians’ time is limited. In such cases, we have found it effective to present preliminary solutions instead of waiting for them to take the initiative, as these serve as concrete starting points that can spark meaningful discussion and engagement, even within the limited time other professionals can dedicate to the project. Another strategy is to allocate part of the budget to hire one or more healthcare professionals specifically for the project. These could be, for example, trained nurses who can efficiently act as mediators between technical and medical teams.

As the project leader, you must do your best to convince all team members about the advantages of an active participation in the project (e.g., patients’ gratitude, increased hospital reputation, increased quality of care, publications). Try to understand the problems of the other team members, because this helps to find solutions and run the project smoothly.

## Rule 4: Be very specific when writing requirements

This is the who-does–what issue. In the project proposal, workpackages provide a detailed overview of the project activities, describing tasks, timelines, and deliverables. They also define the specific roles and responsibilities of each partner, ensuring clarity regarding who is responsible for what and when. However, some project requirements may become clear only later, once the project is already running. Writing requirements without any ambiguities is nearly impossible, particularly during the grant preparation stage. It is therefore essential to collectively establish strategies and tools for early requirements definition. These may include project management frameworks tailored for technical partners, as well as co-design plans jointly developed by technical teams and potential end users. Regular consensus meetings and periodic reviews of requirements are crucial to prevent misunderstandings and to enable early mitigation measures in case of emerging conflicts. We recommend that the project leader sets up a dedicated task force, including technicians, system designers, and representatives of the end users, to oversee the final requirements collection. In fact, what seems very clear to you may be interpreted differently by another team member, especially if you have different backgrounds. For example, software developers who are specialized in financial apps, may have some problems in interpreting requirements of a healthcare application. Additional challenges can arise from technical constraints imposed by hospital IT procedures. In CAPABLE, for example, we discovered only at the time of the clinical study that penetration tests were mandatory in one hospital, which resulted in the need for extra time and money. In general, when a partner realizes they have more tasks than anticipated, they may request additional budget. The primary solution is to redistribute the available resources, as bringing in an additional partner would introduce more bureaucracy and higher costs. There are two options: (i) allow the partner to offload tasks, seeking other consortium members who can fill the gap at no extra cost, or even remove non-essential tasks from the plan; (ii) increase the partner’s budget convincing the others to proportionally contribute. A third option, which should be avoided under any circumstances, is presuming that no partner will revise their budget, thus forcing your group to take on additional tasks without compensation in order to “save” the project. Ultimately, the negotiation will be entirely led by you, the project leader, and the results will depend on your negotiation skills.

## Rule 5: Do expect that somebody will move

During the lifespan of a large project lasting 3–5 years, it is not surprising that some people will leave the project, either moving to another role in the same institution (frequent within companies) or leaving the project partner altogether. When key people leave, it can trigger a domino effect, with colleagues following, potentially escalating the issue to a critical level. As the project leader, you will be the one to manage these challenges. Replacing team members can be challenging, especially if they played a critical role in earlier project phases, as new individuals may have different ideas or expertise. The ease of this transition may also depend on the circumstances of the persons departure, whether amicable or strained with their institution. If amicable, the new institution joined by people who left, can be included in the consortium, in the form of a new partner, a subcontractor, or in-kind contributor. In any case, be prepared to take proactive steps to ensure that institutions are committed to assigning replacements who can fulfill the responsibilities left vacant. Sometimes you may need to adjust some project features. For example, in CAPABLE, when a clinical principal investigator changed, we had to adjust the patient selection criteria for the clinical trial of the project. In fact, especially in small cancer centers, the oncology team expertise impacts on the type of cancer patients who choose to be treated in that center. Another challenge associated with personnel change is ensuring a smooth handover of specific tasks, which brings us back to Rule 4: it is crucial that each requirement is thoroughly documented to ensure the correct interpretation by new team members.

## Rule 6: Be aware of and respect other people’s settings, culture, and habits

In a large project, the consortium typically includes institutions from different countries, and the project leader must take into account their different geographical locations, cultures, customs, and religions. The project may involve countries with different time zones, different timing and duration of vacations and religious holidays, and thus different work schedules. During certain periods, it may be nearly impossible to schedule a meeting due to at least one partner being unavailable. While some EU regions may have more flexible work practices, with people willing to join calls during holidays, in other regions, this is considered unconventional. Cultural differences can also influence interactions with partners, especially at higher hierarchical levels. For instance, it’s likely that reaching directors or senior managers may be more challenging in certain cultures. The project leader must respect and adapt to these cultural nuances. By understanding these differences early on, the project leader can plan the project timeline in a way that accommodates everyone’s schedules, ensuring that tasks are completed effectively while respecting cultural norms.

## Rule 7: Consider that research is not the core business of all the partners

A successful consortium of a digital health project combines academic, industrial, healthcare components, and, possibly, government agencies. However, many academic researchers may not be accustomed to working with companies to co-develop a product, with hospitals to deploy it, and with government agencies to establish policies for adoption. Companies, even when involved in research projects, continue to operate with a business mindset, as their goals and missions are naturally different from those of academia. For example, when system requirements are ambiguous (see Rule 4), companies are likely to adopt the interpretation that demands fewer resources. Additionally, companies have a much more rigorous work schedule than academia. They may have hired a person to develop a project task, and if the task is delayed by other, less constrained, partners, it is a loss to the company. Healthcare organizations also frequently include a significant business component that needs to be considered, even more so in the case of private institutions. Where possible, it is advisable to anticipate potential sources of friction and formalize them during the contract negotiation phases, specifically, in EU-funded projects, when the CA is drafted. This agreement can serve as a valuable tool to establish clear expectations, responsibilities, intellectual property rights, and mechanisms for resolving conflicts among partners. For their part, companies should recognize the benefits of participating in large research projects. They should explore the possible business models to make some profit from maintaining the system after the project is completed. This is very important to avoid the project falling into the “valley of death,” mainly when patients complain about the interruption of what they consider to be a nice and useful service.

## Rule 8: Allow plenty of time and effort for ethics and regulatory activities

Worldwide, funding agencies enforce strict ethical standards in research projects, often requiring specific deliverables and WPs. For clinical studies, a detailed annex is often needed at the time of the proposal submission, and projects may undergo additional reviews. Once the proposal is funded, and the clinical study may start, the Ethical Committees or National Regulatory Bodies might anyway not approve the study protocol, e.g., because regulations on privacy or medical devices changed in the meantime. Additionally, in international projects, regulations can be interpreted differently across countries. For instance, in CAPABLE, the study notification to the national regulatory bodies required more requests and iterations in Italy than in The Netherlands, impacting on the planned timing of activities. Overall preparation for the execution of the clinical pilot studies began in September 2021, with authorizations obtained in January 2023 in the Netherlands and in May 2023 in Italy. The PC must engage with the relevant notified bodies, which may request modifications to the protocol. If the consortium lacks the expertise to address those requirements, you must hire an external expert. For instance, when we were required to apply the UNI EN ISO 14971 standard for risk management in medical devices, we brought in external expertise. Finally, some of the activities foreseen may overlook some regulatory implications, especially when new regulations are introduced. For example, CAPABLE was submitted in 2019, when the GDPR was in its early adoption phase, and the current EU Medical Device Regulation (MDR 245/2017) came into force in 2021, when the project was ongoing. This evolving regulatory scenario impacted the use of a smartwatch, originally selected because of valuable technical features such as blood pressure monitoring. Unfortunately, it was not yet certified as a medical device, which posed challenges for its use in clinical trials, and we had to exclude it from the decision-support functions.

These experiences highlight the importance of careful and realistic planning at the proposal stage. When preparing a clinical study within a digital health research project, it is crucial to allocate adequate time and resources for regulatory and ethical compliance. Estimations should consider the regulations applicable in the countries where the study will take place (typically concerning data protection, clinical investigations involving medical devices, and the use of AI) along with the specific administrative processes of the participating clinical centers and the overall study design. It is also advisable to assess early on whether external support, such as contracting a Clinical Research Organization, may be necessary.

## Rule 9: Do not underestimate dissemination and technology transfer

Often considered as secondary by researchers, these activities are in fact crucial to ensure that the project has an impact beyond its formal duration. As emphasized in previous rules, strategic planning from the proposal-writing stage is essential to sustain such impact. Ideally, the consortium should include a partner specifically dedicated to technology transfer (see Rule 7), particularly in large, multi-partner projects. During the project’s lifetime, additional actions can substantially enhance visibility and long-term impact. These include participating in project clusters and capacity-building initiatives, as well as joining programs promoted by funding bodies, such as the EU’s Horizon Results Booster [11], which provides mentoring services to help EU-funded projects maximize their results and societal impact. Researchers may also contribute to sustainability by looking for further fundings to support the implementation or expansion of some of the project results.

In CAPABLE, the identification of exploitable results and the roadmap for intellectual property protection and management were primarily carried out by the scientific partners, but this required unexpected, significant effort. Considerable resources were also dedicated to keeping the project website (capable-project.eu) up-to-date and attractive, and to promote the project in the local and national press and social networks (e.g., a total of 110 Instagram posts were published in 2023, each featuring content tailored to different target audiences). These actions indeed gave the project great visibility (the Instagram page of the project had 680 followers at the end of 2023), and students from other faculties and universities used CAPABLE as a case study for their thesis. We consider this a general principle. Even in projects primarily focused on basic research, communicating to the public about the potential future impacts of the work helps foster trust in the national and local scientific and healthcare institutions.

**Fig 2:**
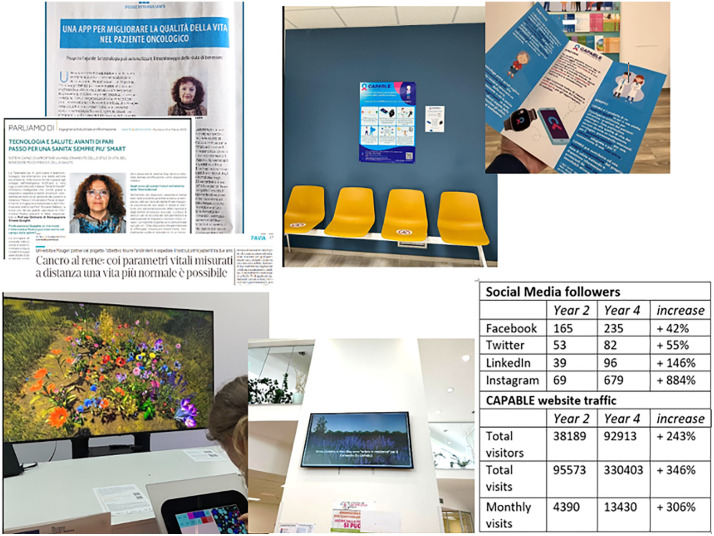
Dissemination activities in the CAPABLE project. Communication through national and local newspapers to inform citizens; promotion inside the hospital; showcase of the Digital Garden artwork; activities on social media (the identifiable person in the picture is one of the authors—SQ. The individual in this photograph has given written informed consent (as outlined in PLOS consent form) to publish this image). Newspaper pictures were reproduced with permission from “CS Communication—Magazine Sanità&Benessere efocus” and “La Provincia Pavese”.

As an innovative dissemination experiment, CAPABLE partnered with two artists-in-residence specialized in technology-focused art. They created an artwork inspired by the CAPABLE drug-drug interaction checker, which helps physicians make safer prescriptions. The artists developed an “AI digital garden,” where each plant represents a drug, and plants grow robustly or wither depending on positive or negative interactions with neighboring plants. It has been showcased in various exhibitions, museums, and cultural events [12]. Fig 2 shows some of the dissemination activities that were performed during the CAPABLE project.

## Rule 10: Plan some activities for users’ engagement and project team building

**Fig 3:**
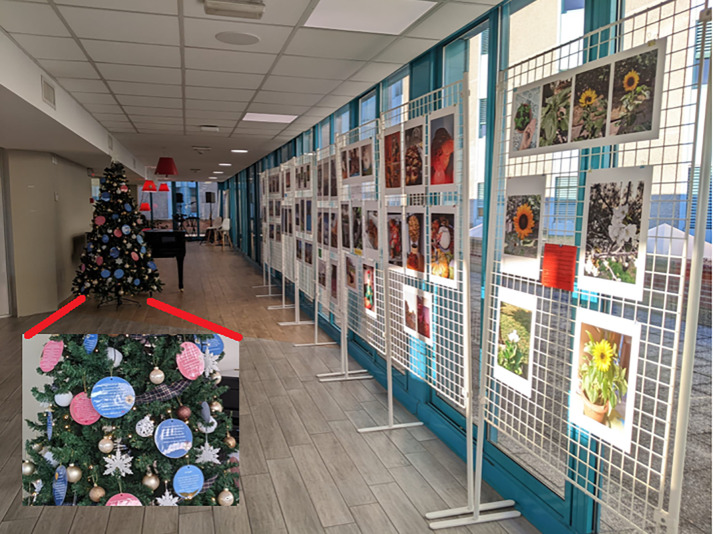
Patients engaging and fun activities: the Christmas party at the hospital. Right: the exhibition of the pictures taken by patients to depict their cancer journey. Left: the tree decorated with patients’ thoughts.

The last rule in this paper recommends to plan some activities to both make patients feel part of an important project and improve the project team cohesion. This often offers an opportunity to create moments of relaxation, as with a bit of creativity it is possible to organize enjoyable activities that can also help promote the project externally. For example, in CAPABLE, the patient app allowed users to take photos and to add captions that reflected their current mood. Patients volunteered to share this material, and we curated a photo exhibit at the annual Christmas Party of Cancer Patients organized at the Maugeri Hospital in Pavia (Fig 3). For the same event, we decorated the Christmas tree with ornaments displaying messages from patients collected through the app’s feature “Vase of Gratitude,” where patients could write dedications to friends, family, and others. The photographic exhibition attracted around 100 attendees on the opening day and remained on display for one month. In total, 147 photographs were collected, taken by 24 patients. We also organized focus groups to discuss nutritional and psychological issues, creating a supportive and relaxing space for deeper engagement.

Beyond the described specific examples of activities, inspired by the CAPABLE app’s functionalities, any project in this field that involves patients may set up initiatives to engage its users, who may include patients, their home caregivers, or healthcare personnel.

Team-building activities were also carried during the project meetings, guided by the idea that a strong and cohesive team is better prepared to face the project challenges. During in-person meetings, we focused on cooking, an activity that has been shown to strengthen interpersonal relationships [13]. The kickoff meeting in Rome included a dinner session where participants learned how to make pizza, a fun experience particularly for the many international partners. Similarly, during a meeting in Amsterdam, we participated in a cooking workshop centered on Dutch flavors at a local cooking studio. During online meetings, which, in our case were necessitated by the COVID-19 pandemic, we made use of online tools that became widely adopted in those years [14,15].

## Discussion

In this section, we reflect on some lessons learnt and limitations. From our experience, drawn not only from CAPABLE but also from other similar research projects, technological transfer remains one of the primary challenges. Too often, results remain confined to laboratories, and after pilot studies, the developed systems are abandoned. Regarding the efforts related to sustain the CAPABLE project also after its official end, in 2023, we joined the CS_AIW (Cancer Survivorship—AI for wellbeing) cluster of projects [16], guided by the FAITH H2020 consortium. This cluster was designed to foster cross-fertilization and shared learning among project experiences. CAPABLE also participated in a series of three workshops organized by the Horizon Results Booster initiative, which provided an opportunity to identify actionable strategies for ensuring the project’s long-term sustainability. Unfortunately, no partner within the consortium took the lead to transform CAPABLE into a commercial product, partly due to the complexity of the multi-partner technological development.

A key lesson from this experience is that sustainability activities should be planned at the time of consortium formation to maximize their effectiveness over the project’s lifetime. Despite this, the team pursued alternative routes to sustain its outcomes. After some unsuccessful attempts to reconstitute part of the consortium for larger follow-up studies, new funding was eventually secured for two projects, thereby continuing the legacy and applied impact of CAPABLE. The first project, funded in 2023 by the National Recovery and Resilience Plan (PNRR-TR1-2023-12377022), is centered on testing the efficacy of prehabilitation on the pathway of patients suffering from head and neck cancer and candidate to surgery or chemoradiotherapy. The second project is called WISH (Women’s Integrated Support for Hormonal Health), funded by the European Partnership on Transforming Health and Care Systems in 2025 and that will start in April 2026, which aims to improve the follow-up of breast cancer patients receiving hormonal therapy and integrates an app for symptoms and adverse events monitoring.

As also reflected in our rules, another important lesson is not to underestimate activities related to regulatory compliance and constraints. The recommended approach is to allocate adequate budget and personnel, and to select partners with the appropriate expertise to carry out this work, particularly in the context of an evolving regulatory environment, which is common in highly innovative projects. In CAPABLE, regulatory requirements represented one of the main challenges encountered.

While the rules presented in this paper are general, we acknowledge that our experience is primarily based on European projects involving public research institutions. Experiences related to technology transfer and other project aspects may differ in other funding environments, particularly those oriented toward public-private partnerships.

## Conclusion

The above rules reflect what has been our approach in coordinating the CAPABLE project. In Rule 1, we have highlighted that effective coordination relies on assembling motivated teams, balanced in terms of skills (technical, medical, legal), geographical location, and gender. Rule 2 recommends securing effective support for the organizational management of the project. If this is not available within the leading institution, it’s useful to hire a skilled PM, who will also help with communication with the funding agency. Rule 3 reinforces the leadership role of the PC, who should indeed seek the support of the other partners, but must always be ready to personally address crucial decisions and steer the team in the right direction. Rule 4 reminds us of the IT dimension of the projects we are dealing with, namely the importance of collecting functional and technical requirements as precisely as possible, to avoid misunderstandings regarding technical development responsibilities, which could also have an impact on the budget. Rule 5 advises being prepared for the possibility that somebody may leave the project, as this is a natural occurrence in projects that last several years. Rule 6 reminds us that clear communication and mutual respect across disciplines, institutions, ways of working, and cultures are essential to ensure smooth collaboration. Rule 7 aims to prevent potential conflicts arising from the fact that research is not always the primary business of a partner, for example, a company that must adhere to profit-oriented behaviors. Rule 8 brings our attention to ethical and regulatory aspects, which must be integrated from the project planning phase. Rule 9 outlines the importance of strategic planning for dissemination, exploitation, and sustainability beyond the project’s duration. Finally, Rule 10 suggests a set of activities for team building and user engagement.

We believe that these rules support a project management that is both decisive and collaborative, guiding the team toward shared goals with clarity and cohesion.

## References

[1] Ward CL, Shaw D, Sprumont D, Sankoh O, Tanner M, Elger B. Good collaborative practice: reforming capacity building governance of international health research partnerships. Global Health. 2018;14(1):1. doi: 10.1186/s12992-017-0319-4 29310698 PMC5759302

[2] Morrison M, Mourby M, Gowans H, Coy S, Kaye J. Governance of research consortia: challenges of implementing Responsible Research and Innovation within Europe. Life Sci Soc Policy. 2020;16(1):13. doi: 10.1186/s40504-020-00109-z 33190636 PMC7667809

[3] World Health Organization. Global strategy on digital health 2020–2025. Geneva: WHO; 2021.

[4] Patanakul P. Managing large-scale IS/IT projects in the public sector: problems and causes leading to poor performance. J High Technol Manag Res. 2014;25(1):21–35. doi: 10.1016/j.hitech.2013.12.004

[5] Brocke J vom, Lippe S. Managing collaborative research projects: a synthesis of project management literature and directives for future research. Int J Project Manag. 2015;33(5):1022–39. doi: 10.1016/j.ijproman.2015.02.001

[6] Peleg M, Shahar Y, Quaglini S, Broens T, Budasu R, Fung N, et al. Assessment of a personalized and distributed patient guidance system. Int J Med Inform. 2017;101:108–30. doi: 10.1016/j.ijmedinf.2017.02.010 28347441

[7] Fraterman I, Wollersheim BM, Tibollo V, Glaser SLC, Medlock S, Cornet R, et al. An eHealth App (CAPABLE) providing symptom monitoring, well-being interventions, and educational material for patients with melanoma treated with immune checkpoint inhibitors: protocol for an exploratory intervention trial. JMIR Res Protoc. 2023;12:e49252. doi: 10.2196/49252 37819691 PMC10600650

[8] Dagliati A, Sacchi L, Tibollo V, Cogni G, Teliti M, Martinez-Millana A, Traver V, Segagni D, Posada J, Ottaviano M, Fico G, Arredondo MT, De Cata P, Chiovato L, Bellazzi R. A dashboard-based system for supporting diabetes care. J Am Med Inform Assoc. 2018 May 1;25(5):538–47. 29409033 10.1093/jamia/ocx159 PMC7647008

[9] European Commission D-G for R and I. Funding and tenders portal – survey report. 2022.

[10] APRE. Horizon Europe implementation guide; 2023. Available from: https://apre.it/wp-content/uploads/2023/06/horizon-europe-implementation-KI0323236ENN.pdfAPRE

[11] Horizon Results Booster [cited 14 Nov 2025]. Available from: https://www.horizonresultsbooster.eu/

[12] Dumitriu A. Capable project residency [cited 2025 Nov 14]. Available from: https://annadumitriu.co.uk/portfolio/capable-project-residency

[13] Howell CD, Willcox LA. A recipe for successful collaboration: shared creative work experiences (SCrWE) among co-researchers | Ein Rezept für erfolgreiche Zusammenarbeit: shared creative work experiences (SCrWE) in Forschungsteams. Forum Qualitative Sozialforschung. 2024;25(2):4.

[14] Carruthers R. 34 virtual team building activities for remote employees. Together Platform; 2021. Available from: https://www.togetherplatform.com/blog/10-virtual-team-building-activities-for-your-remote-employees

[15] Virtual team building [cited 2025 Dec 7]. Available from: https://www.virtualteambuilding.eu/

[16] CS_AIW (Cancer Survivorship – AI for wellbeing) cluster of projects [cited 14 Nov 2025]. Available from: https://www.cs-aiw.eu/

